# Hemispheric lateralization of a molecular signal for pain modulation in the amygdala

**DOI:** 10.1186/1744-8069-4-24

**Published:** 2008-06-23

**Authors:** Yarimar Carrasquillo, Robert W Gereau IV

**Affiliations:** 1 Washington University Pain Center, Department of Anesthesiology, Washington University School of Medicine, 660 S. Euclid Ave., Campus Box 8054, St. Louis, MO, 63110, USA; 2 Department of Developmental Biology, Washington University School of Medicine, St. Louis, MO 63110, USA

## Abstract

The extracellular signal-regulated kinase (ERK) cascade has been shown to be a key modulator of pain processing in the central nucleus of the amygdala (CeA) in mice. ERK is activated in the CeA during persistent inflammatory pain and this activation is both necessary and sufficient to induce peripheral tactile hypersensitivity. Interestingly, biochemical studies show that inflammation-induced ERK activation in the CeA only occurs in the right, but not the left hemisphere. This inflammation-induced ERK activation in the right CeA is independent of the side of peripheral inflammation, suggesting that there is a dominant role of the right hemisphere in the modulation of pain by ERK activation in the CeA. However, the functional significance of this biochemical lateralization has yet to be determined. In the present study, we tested the hypothesis that modulation of pain by ERK signaling in the CeA is functionally lateralized. We acutely blocked ERK activation in the CeA by infusing the MEK inhibitor U0126 into the right or the left hemisphere and then measured the behavioral effects on inflammation-induced mechanical hypersensitivity in mice. Our results show that blockade of ERK activation in the right, but not the left CeA, decreases inflammation-induced peripheral hypersensitivity independent of the side of peripheral injury. These findings demonstrate that modulation of pain by ERK signaling in the CeA is functionally lateralized to the right hemisphere, suggesting a dominant role of the right amygdala in pain processing.

## Findings

The amygdala is a forebrain multinuclear structure with a well-established role in emotional processing [1,2]. Increasing evidence supports the role of the central nucleus of the amygdala (CeA) as a neural modulator of pain perception [3]. Previous work from our laboratory has identified the extracellular signal-regulated kinases (ERKs) as key molecules for the modulation of pain by the CeA in mice [4]. Biochemical experiments showed that ERK is activated in the CeA during persistent inflammation. ERK activation in the CeA was shown to be necessary for inflammation-induced peripheral hypersensitivity because acute pharmacological blockade of ERK activation in this amygdala nucleus reduced inflammation-induced peripheral tactile hypersensitivity. Furthermore, ERK activation in the amygdala was shown to be not only necessary for inflammation-induced peripheral hypersensitivity but also sufficient to induce peripheral tactile hypersensitivity in the absence of tissue injury.

Interestingly, the biochemical data from our previous study showed that inflammation-induced ERK activation occurs in the right CeA independent of the side of peripheral inflammation (right or left hindpaw) [4]. These results suggest that modulation of pain by ERK activation in the CeA might be functionally lateralized to the right hemisphere. We tested this hypothesis in the present study by comparing the effects of acute blockade of ERK activation in the right versus the left amygdala when inflammation was induced in the right or the left hind-paw.

To induce peripheral inflammation in mice, a 5% formalin solution was injected subcutaneously into the right or the left hind-paw as previously described [4]. Two hours after formalin injection into the hind-paw, the MEK inhibitor U0126 (1.5 nmoles), the inactive structural analog U0124 (1.5 nmoles) or vehicle (50% DMSO/Saline) were infused into the right or the left CeA. Formalin-induced mechanical sensitivity was measured 1 hr after intra-amygdala drug infusion, which corresponds to 3 hrs after formalin injection into the hind-paw. This time-point was selected based on the results from our previous study that show that ERK is activated in the CeA 3 hours after formalin injection into the hind-paw [4]. At this time-point we also observed significant formalin-induced mechanical hypersensitivity, in both the injected and the uninjured contralateral hind-paw [4].

**Figure 1:**
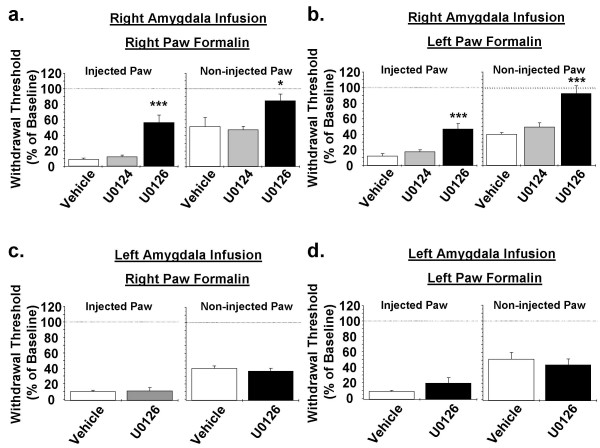
**Effects of intra-amygdala U0126 infusion on formalin-induced mechanical hypersensitivity.** Five days after cannulation, animals were injected with 5% formalin into the right (**a**, **c**) or the left (**b**, **d**) hind-paw. Two hours after the paw injection, mice were infused into the right (**a**, **b**) or the left (**c**, **d**) amygdala with the MEK inhibitor U0126 (n = 6 animals), the structural analog control compound U0124 (n = 6 animals) or vehicle (n = 5–6 animals). One hour after the amygdala injection, the effects of these treatments on mechanical thresholds were analyzed. U0126 infusion into the right amygdala significantly reduced formalin-induced primary hypersensitivity and eliminated secondary hypersensitivity, independent of the side of paw injection (p < 0.05, One-way ANOVA, Tukey's post-test; * = p < 0.05, *** = p < 0.0001) (**a**, **b**). U0126 infusion into the left amygdala did not significantly affect formalin-induced mechanical hypersensitivity (**c**, **d**). Data displayed in panel A was obtained with permission from Carrasquillo and Gereau, J Neurosci. 2007 Feb 14;27 (7): 1543–51.

In our previous study, we induced peripheral inflammation in the right hind-paw and performed the intra-amygdala drug infusions in the right amygdala. Under these experimental conditions, blockade of ERK activation in the right CeA significantly decreased formalin-induced mechanical hypersensitivity, both in the injected paw and in the uninjured contralateral paw, compared to animals injected with vehicle or U0124 (Fig 1a) [4]. In the present study, we tested whether ERK activation in the right CeA modulates pain independently of the side of peripheral inflammation by injecting formalin into the left hind-paw and then infusing U0126, U0124 or vehicle into the right CeA. Blinded behavioral analysis revealed that, similar to our previous results in the right paw-injected mice, the left paw-injected mice that received an infusion of U0126 into the right CeA also exhibited significantly reduced formalin-induced mechanical hypersensitivity in both the injected and the uninjured contralateral hind-paw (Fig 1b). Thus, mechanical thresholds in the injected paw of U0126-infused mice were 0.63 g ± 0.12 (mean ± se; approximately 47% of baseline); while vehicle- and U0124-infused mice had mechanical thresholds of 0.10 g ± 0.02 and 0.16 g ± 0.00 (mean ± se; approximately 15% of baseline) respectively. Similar effects on mechanical thresholds were observed in the uninjured contralateral hind-paw, with a mean threshold of 0.97 g ± 0.13 (mean ± se; approximately 93% of baseline) in U0126-infused mice and 0.47 g ± 0.04 (mean ± se; approximately 45% of baseline) in both vehicle- and U0124-infused mice. Consistent with our previous study, intra-amygdala infusion of the structural analog U0124 did not significantly affect formalin-induced primary or secondary hypersensitivity as mechanical thresholds in the injected and uninjured contralateral hind-paw where not significantly different in vehicle- and U0124-infused mice. The results from the present study where we induce inflammation in the left hind-paw, together with the results from our previous work where we induced inflammation in the right hind-paw [4], show that inhibition of ERK activation in the right CeA significantly reduces formalin-induced mechanical hypersensitivity independent of the side of peripheral inflammation. These behavioral results nicely correlate with our previous biochemical data that showed significant formalin-induced ERK activation in the right amygdala independent of the side of hind-paw injection [4]. Also consistent with our previous findings, mechanical thresholds in the uninjured contralateral hind-paw of U0126-infused mice were not significantly different from pre-infusion baseline mechanical thresholds (1.03 ± 0.08; mean ± se), suggesting that blockade of ERK activation in the right CeA completely eliminates formalin-induced secondary hypersensitivity.

Since the biochemical data from our previous work showed a strong formalin-induced ERK activation in the right, but not the left amygdala [4], we next asked whether the effects of blockade of amygdala ERK activation are specific to the right amygdala or if inhibiting ERK activation in the left amygdala would have similar behavioral effects. To do this, we infused the MEK inhibitor U0126 or vehicle into the left amygdala and then measured the effects on formalin-induced mechanical hypersensitivity in both the injected and the non-injured contralateral hind-paw. Blockade of ERK activation in the left amygdala had no significant effect on formalin-induced primary or secondary hypersensitivity whether formalin was injected into the right or the left hind-paw (Fig 1c and 1d). These experiments demonstrate that modulation of pain perception by amygdala ERK activation is preferentially mediated by the right amygdala, suggesting a dominant role of the right amygdala in pain processing.

Altogether, our results show a clear hemispheric lateralization of a central component of pain processing in the amygdala of mice. Thus, blockade of ERK activation in the right but not the left amygdala decreased inflammation-induced hypersensitivity independently of the side of peripheral inflammation. Hemispheric lateralization of amygdala function has been reported in humans and in rodents [5-16]. Most of these studies measured neural activity changes related to emotional memory and report differential activation of the left and the right amygdala in humans during this process [5-10]. While there is no general agreement on the reasons for this lateralization, it has been proposed to be dependent on the valence of the stimulus (negative versus positive) [5]; on whether the experience is conscious or unconscious [6]; learned through anticipation or by actual experience [7]; or due to gender differences [8-10]. Particularly relevant to our findings, fMRI studies in patients with gastric pain have also shown lateralized amygdala activation in humans, with increased neural activity observed in the right amygdala during gastric pain [11]. In rodents, although only scarcely reported, studies have also described lateralization of amygdala function in emotional learning and memory processes, with the right amygdala playing a dominant role [12-16]. These reports may be scarce because most animal studies of amygdala function have evaluated the two amygdalae together and as functionally equivalent.

Anatomical studies using retrograde tracers have identified projections from the CeA to the periaqueductal gray (PAG) [17]. During long-term stress, increased neural transmission has been observed in this CeA-PAG pathway in rats [18,19]. Interestingly, consistent with our data, these changes in neural transmission during long-term stress occur in the right, but not the left hemisphere. It is therefore possible that ERK activation in the right CeA modulates pain by modulating synaptic transmission in the CeA-PAG pathway, which might in turn feed into the PAG-rostral ventromedial medulla-dorsal horn descending modulatory pain pathway. However, whether ERK activation in the CeA occurs in neurons that project to the PAG remains to be determined.

To our knowledge, this is the first study that shows a functional hemispheric lateralization of a central component of pain processing in the amygdala of mice. Our findings thus raise many interesting questions that are yet to be determined: Is this hemispheric lateralization specific to inflammatory pain or is it also seen in other types of pain? Is this a molecular or an anatomical lateralization? What is the mechanism underlying this lateralization? What is the functional significance of and the anatomical explanation for this hemispheric lateralization? Although our study does not help to clarify these issues regarding lateralization, it does provide an animal model that can be exploited for future investigations into these questions.

## Methods

### Subjects

Experiments were performed in accordance with the guidelines of the NIH and were approved by the Animal Care and Use Committee of Washington University School of Medicine. Male Swiss-Webster mice (40–45 g) were housed in 12 hr light/dark cycles with food and water provided ad libitum.

### Surgical procedure

Mice were deeply anesthetized with a combination anesthetic (2.5 ml/kg body weight) consisting of Ketamine (37.5 mg/ml), Xylazine (1.9 mg/ml), and Acepromazine (0.37 mg/ml) and were chronically implanted with an 8 mm 26-G stainless steel guide cannula aimed at the central nucleus of the amygdala (stereotaxic coordinates used: 1.4 mm posterior from bregma, 3.3 mm lateral to midline, 4.2 mm ventral from skull surface). The guide cannula was fixed to the skull using 2 jeweler's screws and dental acrylic. An 8 mm stylet was placed in the cannula to prevent clogging. Animals recovered for at least one week before additional experimental procedures. At the end of the experiment, brains were sectioned and nissl stained to verify cannula position and injection site.

### Intra-amygdala drug infusions

Microinjections were performed through a 32-G stainless steel injection cannula that extended 0.5 mm beyond the tip of the guide cannula. The injection cannula was attached to flexible plastic tubing and a microliter Hamilton syringe to perform the injections. A total volume of 0.3 μl was infused over a period of 3 min and the injection cannula was kept in place for an additional 1 min to allow for drug diffusion. U0126 and U0124 (VWR Scientific Products) were dissolved in 100% DMSO to a final stock concentration of 10 mM. On the day of the experiment, U0126 and U0124 were diluted 1:1 in 0.9% Saline to a concentration of 5 mM in 50% DMSO/Saline. 50% DMSO/Saline was used as a vehicle control for all experiments. The infused volume and doses of U0126 and U0124 were selected based on our previous biochemical experiments that showed that inhibition of ERK activation by U0126 under these conditions is restricted to the CeA and that 1.5 nmoles of U0126 is the lowest dose that significantly inhibits formalin-induced ERK activation in the CeA [4].

### Nociceptive testing

Mechanical sensitivity was measured using von Frey filaments (North Coast Medical, Inc. San Jose, CA) as previously described [4]. Three to five baseline measurements were taken from each hind-paw before formalin injection and the average was calculated individually for each paw as the baseline. Once the baseline was established, ten microliters of 5% formalin solution was injected subcutaneously into the plantar surface of the right or the left hind-paw. Intra-amygdala drug infusions were performed 2 hrs after formalin injection and mechanical hypersensitivity was measured again 1 hr after intra-amygdala drug infusion, which corresponds to 3 hrs after formalin injection in the hindpaw. Mechanical hypersensitivity was calculated as the percentage of baseline for each hindpaw. All behavioral testing was performed blind to pharmacological treatment.

## Authors' contributions

YC and RWG participated equally in the conception, design, and interpretation of the study. Additionally, YC carried out the experiments, performed the data analysis and drafted the manuscript. All authors read and approved the final manuscript.
